# SMART-ER: a Situation Model of Anticipated Response consequences in Tactical decisions in skill acquisition — Extended and Revised

**DOI:** 10.3389/fpsyg.2014.01533

**Published:** 2015-01-06

**Authors:** Markus Raab

**Affiliations:** ^1^School of Applied Science, London South Bank UniversityLondon, UK; ^2^Performance Psychology, Institute of Psychology, German Sport UniversityCologne, Germany

**Keywords:** embodied cognition, sport, top–down process, bottom–up process, skill acquisition

## Abstract

Situation Model of Anticipated Response consequences in tactical decisions (SMART) describes the interaction of top–down and bottom–up processes in skill acquisition and thus the dynamic interaction of sensory and motor capacities in embodied cognition. The empirically validated, extended, and revised SMART-ER can now predict when specific dynamic interactions of top–down and bottom–up processes have a beneficial or detrimental effect on performance and learning depending on situational constraints. The model is empirically supported and proposes learning strategies for when situation complexity varies or time pressure is present. Experiments from expertise research in sports illustrate that neither bottom–up nor top–down processes are bad or good *per se* but their effects depend on personal and situational characteristics.

## INTRODUCTION

Consider the soccer goalkeeper’s simple task of preventing a penalty shooter scoring a goal. The goalkeeper’s behavior provides a good example of sensorimotor interaction, that is, the interaction of sensory and motor capacities. Given the distance of the ball to the goal (11 m or 12 yards), the mean speed of a ball of more than 20 m/s (Farina et al., 2013), and a required response of the goalkeeper of 100 ms or more before the actual kick (Farina et al., 2013), the goalkeeper must quickly decide which way to go. In simple terms, the goalkeeper’s options are to move to the left, right, or middle. But how can one explain a specific choice—say, a move to the left—and predict when the goalkeeper will jump? The embodied cognition framework suggests that this action is based on immediate and stored sensorimotor experiences.

Sensorimotor interaction has long been described as a sequential and independent process through which an organism perceives a stimulus, cognitively processes that information, and then selects a response (Proctor and Vu, 2006). In the last decades, however, perception and action have been much more tightly linked, as reflected, for instance, in the theory of common coding (Hommel et al., 2001), which assumes that perception and action share common processes and representations. The premise that actions are coded in terms of their anticipated sensory consequences is a principle as old as psychology itself (James, 1890). A goalkeeper activating an action plan anticipates the sensory consequences of a movement when jumping to one of the goal’s corners. Following previous investigations by Beilock (2008) and Pizzera and Raab (2012) developed new predictions about the interaction of sensorimotor and cognitive processes. They suggested that stored sensorimotor experiences can influence cognitive judgments. For instance, umpires may judge observed movements better when they rely on their own sensorimotor system, that is, when they have experience with the movement they are being asked to judge.

In this paper I present a model that describes top–down and bottom–up processes of skill acquisition and their interactions over time and explore how learning shapes the use of these processes. This model follows other dual-process models accentuating the importance of motor activity (e.g., Strack and Deutsch, 2004) and common principles of intuitive and deliberative judgments (Kruglanski and Gigerenzer, 2011). A high level of cognitive control of sensory processing characterizes top–down processes. In the penalty example, cognitive control could use knowledge about, say, the shooter’s preference to shoot to the left corner. Top–down processes are likely to influence the gaze and the interpretation of sensory information. Bottom–up processes are characterized by an absence of cognitive control in sensory processing and use present information more directly. In the penalty example, this could be the position of the shooter relative to the ball (Savelsbergh et al., 2010).

### DYNAMIC INTERACTIONS IN EMBODIED COGNITION

**FIGURE 1:**
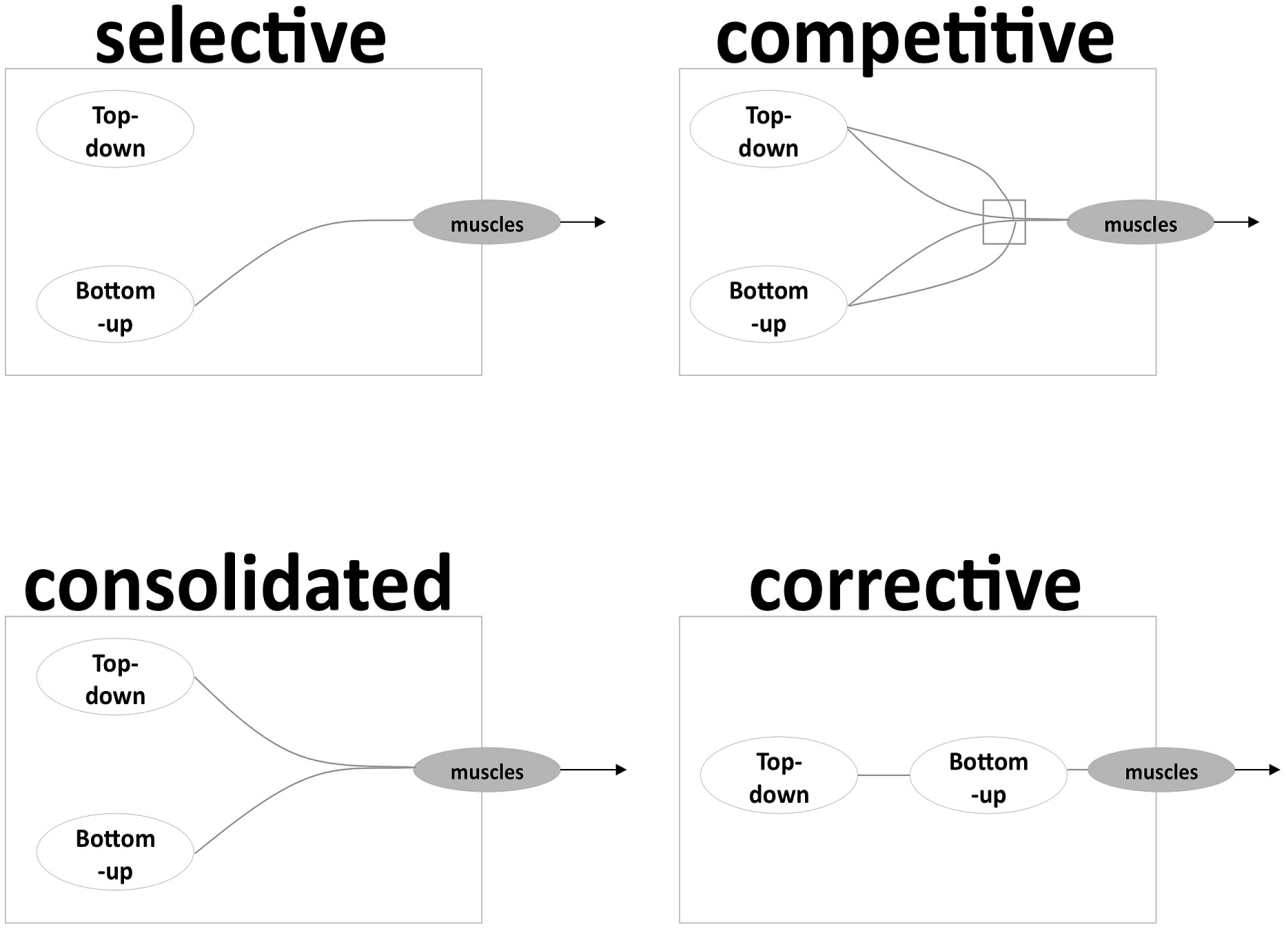
**Four types of interactions of top–down and bottom–up processes in embodied cognition**.

There are at least four different ways top–down and bottom–up processes interact over time (see **Figure 1**). *Selective* interaction means following either top–down or bottom–up processes (i.e., no interaction), so in the penalty example, the goalkeeper would decide to jump to the left based only on knowledge of the shooter’s preferred shooting direction, or the shooter would decide to shoot to the left side independent of the goalkeeper’s moves. This kind of pure selection seems unlikely from an embodied account of cognition (Myachykov et al., 2013). In *competitive* interaction both processes contribute but one process dominates. For example, the goalkeeper would choose to use knowledge of the shooter’s preference to shoot – from the perspective of the shooter – left to decide to jump to his – from the perspective of the goalkeeper – right corner. *Consolidated* interaction means that both processes are involved in the choice. For example, if the top–down process indicates jump left and the bottom–up process indicates jump right, that choice conflict produces displacement activity or frozen behavior (Troisi, 2002); if both processes point in the same direction faster responses occur. *Corrective* interactions describe sequential effects of processes. For example, the goalkeeper may recall the shooter’s preferences and prepare an action tendency toward that preference. However, when observing the shooter approach the ball, the ultimate choice may depend more on bottom–up processes that is for instance the position of the foot relative to the ball. These interactions are dynamic and may depend on previous experience on how success was experienced. Therefore tactical decisions in skill acquisition are embodied in the sensorimotor system and determine how situations are perceived.

### IMPLICITLY AND EXPLICITLY LEARNED SENSORIMOTOR INTERACTIONS

Expert athletes such as the goalkeeper in the penalty example are often defined as having 10 years and about 10,000 h of training that can influence their current choices at any moment in time (Ericsson and Lehmann, 1996). Therefore and to understand the dynamics of sensorimotor interactions within an embodied cognition framework interactions of cognitive and sensorimotor processes need to be modeled with specific types of learning. Learning is often differentiated as implicit and explicit; both types are frequently cited in the motor and cognitive learning literature and researchers largely agree on the definition of the concepts (Kleynen et al., 2014).

Implicit learning is defined as a “non-intentional, automatic acquisition of knowledge about structural relations between objects or events” (Frensch, 1998, p. 76), and explicit learning as an intentional acquisition that results in verbalizable knowledge (O’Brien-Malone and Maybery, 1998). Looking at the learning situation itself in terms of where it sits on the continuum of intentionality may help identify implicit and explicit learning. Situations in which actions are incidental in nature engender implicit learning, whereas situations in which actions are intentional in nature engender explicit learning. In the penalty example, a player learning soccer at the beach may differ from a player in an early selection training group in which a coach and verbalized information about options are available. The former may have learned soccer implicitly and would refer to experienced bottom–up processes more than the latter, who has acquired verbalizable knowledge and may use top–down processes more in subsequent behavior. The interactions of top–down and bottom–up processes might fall into the categories described above. People probably combine years of implicit and explicit learning and thus experience hybrid learning (Mathews et al., 1989). It is unclear if hybrid learning leads to better choices than those made by implicit or explicit learning alone.

## THE EXTENDED AND REVISED SMART: NOW SMART-ER

**FIGURE 2:**
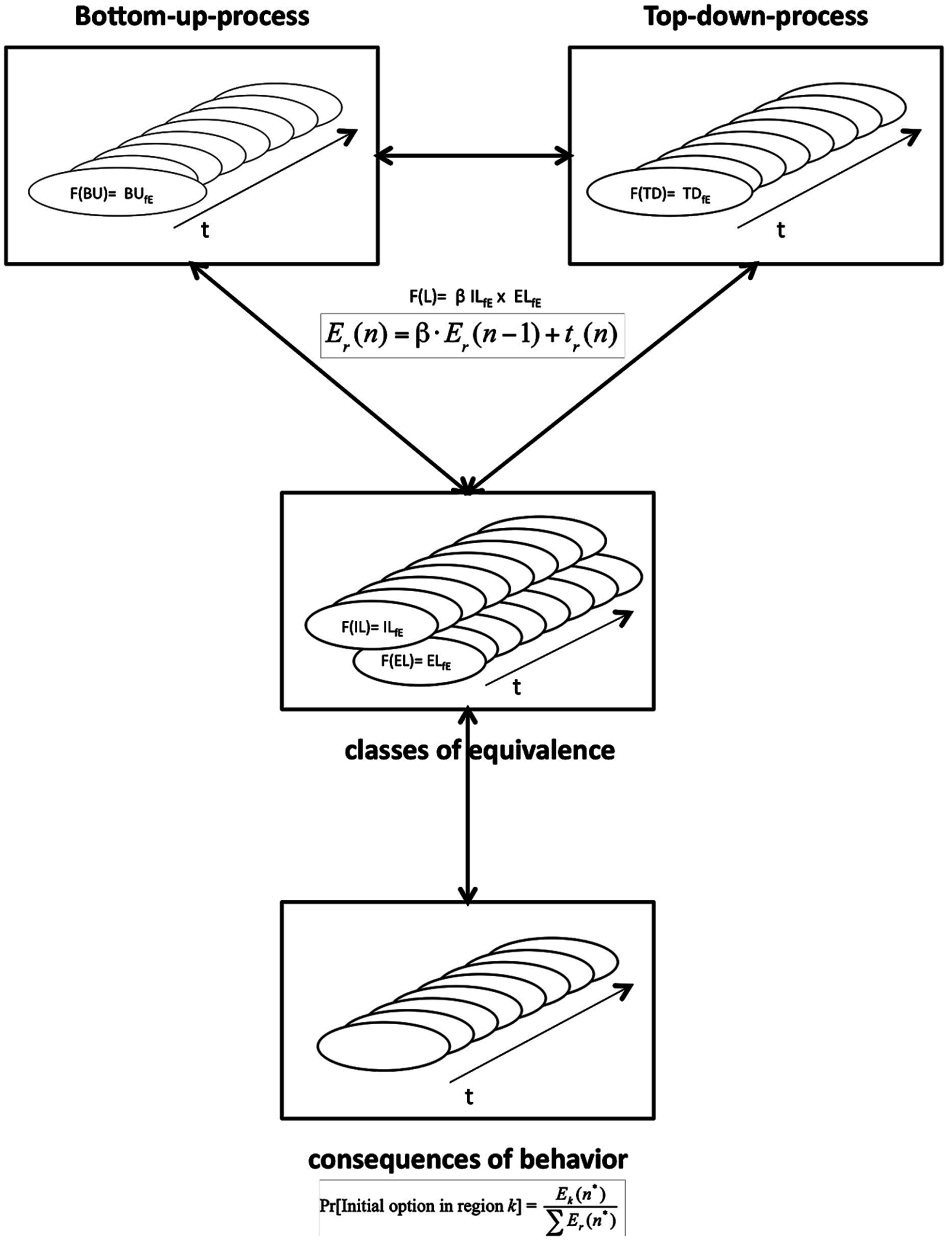
**Situation Model of Anticipated Response-consequences in tactical decisions—extended and revised (SMART-ER).** Top of the Figure Bottom–up (BU) and Top–down (TD) processes are displayed as functions of experience represented in classes of equivalence (fe). Learning is differentiated as Implicit (IL) and Explicit (EL) learning. β represents weighting of implicit and explicit learning over time of accumulated experience. Evidence (E) of a specific region (r) when perceiving information in the environment is used in a choice rule, in which the initial option in a specific region (k) is contrasted to later evidence in other specific regions (r). Details for equations for weighting top–down and bottom–up processes can be found in Glöckner et al. (2012).

One model that explicitly predicts the effects of implicit and explicit learning on anticipated response consequences of actions is SMART, the Situation Model of Anticipated Response consequences in tactical decisions (Raab, 2007), in which time-pressure decisions in sports (e.g., a tactical choice of shooting or passing) are explained as a function of the interaction of top–down and bottom–up processes. In this model (see **Figure 2**), these processes dynamically interact as described above but in addition, a representation format, or equivalence class (Hoffmann et al., 2004), is chosen. Equivalence classes are representations of sensorimotor interactions of anticipated response consequences. An anticipated response consequence describes a representation of the sensorimotor system in which we predict future (anticipated) changes in the environment as a consequence of our movements. Equivalence classes are representations of sensorimotor interactions active when anticipating response consequences that group the consequences of specific choices together. Finally previous implicitly or explicitly learned behavior activates a choice rule that allows one to accumulate information before choosing between certain options (Glöckner et al., 2012).

Here I present an extended (E) and revised (R) SMART, and thus it is labeled SMART-ER. An important extension is the additional focus beyond the person on situations in which dynamic situation-specific sensorimotor interactions take place. Such situations are characterized by whether a fixed set of options are present, as has been used in most research to validate SMART (Raab, 2007), or whether people generate different options themselves, as demonstrated in option-generation paradigms (Johnson and Raab, 2003). SMART has also been extended to include specific predictions based on the complexity of a situation, which can be manifested in the number of choice options, the visual information available, and the speed at which decision have to be made (Raab, 2003). SMART has been revised to specify when implicit learning and explicit learning—in contrast to hybrid learning—may be beneficial. SMART’s predictions of when implicit motor learning is beneficial have been revised to be valid in less complex situations in which the sensorimotor interactions do not require attention regulation via top–down processes. In complex situations, explicit motor learning may be beneficial because it uses knowledge to attend to “information-rich” areas (Magill, 1998). Finally, hybrid learning may be beneficial in complex situations because this type of learning allows the interaction of top–down and bottom–up processes to be calibrated during learning. In simple situations, in contrast, bottom–up processes could regulate the choice and top–down processes would interfere and potentially deteriorate performance.

In the following sections I present empirical evidence supporting SMART-ER. Evidence has been found in laboratory research on the complex movements involved in choices in team sports (Raab, 2003; Raab et al., 2005). In other work, manipulation of top–down and bottom–up processes were tested by applying time-pressure, reducing access to knowledge from long-term memory (Raab, 2003). Instruction manipulations have also been applied (Raab and Masters, 2009). Measures of bottom–up processes include the percentage of early fixations to areas in which the final choice is present (Raab and Johnson, 2007), and for top–down processes whether the first option generated will be overruled if more time for generation is given (Johnson and Raab, 2003). Response time and gaze data have been used to predict participants’ first and final choices and model predictions have been cross-validated to other trials or samples (Glöckner et al., 2012).

### EMPIRICAL EVIDENCE OF TOP–DOWN AND BOTTOM–UP PROCESSES

Based on the tradition separating dual-processes (Chaiken and Trope, 1999) and the above-cited specific models from Strack and Deutsch (2004), Beilock (2008), and Kruglanski and Gigerenzer (2011) my own work supports these notions using longitudinal data. For instance, in a longitudinal study, expert team-handball players were asked to identify the best option for a playmaker in a video displaying attack situations. The task assessed (a) participants’ first choices, (b) alternative options participants deemed appropriate, and (c) after all options were generated, the option participants chose as the best one (Raab and Johnson, 2007). Decision time and gaze behavior, indicating fixations on these options, were measured. Experts abandoned their first option in about 40% of cases and chose a different best option, possibly indicating a top–down influence, as the visual display did not change. Bottom–up processes have been identified by early fixations to important options (Raab and Johnson, 2007).

### EVIDENCE OF TOP–DOWN AND BOTTOM–UP PROCESS INTERACTIONS

In the above-described study (Raab and Johnson, 2007), systematic gaze behavior (e.g., fixation on options on the left attack side) became more strongly correlated with the generation strategy (e.g., generating options on the left side) over the course of the study, indicating consolidated interactions of top–down and bottom–up processes. Measuring fixations over time makes it possible to predict the weighting of early and late information. Results of a study by Raab and Laborde (2011) showed best model fits and cross-validation of model predictions for two-thirds of the participants when early information was weighted more than late information (i.e., indicating less reliance on top–down processes). For the remaining third of participants the pattern was reversed, and thus individual differences in how much deliberation using top–down processes is needed before a final choice is made may explain these patterns.

### EVIDENCE OF SITUATION-SPECIFIC LEARNING EFFECTS

Situation Model of Anticipated Response consequences in tactical decisions – extended and revised predicts situation complexity affects implicit and explicit as well as hybrid learning. Results from four experiments with option-selection tasks in three different sports indicate that indeed, implicit learning produces better and faster choices when manipulating low complex situations (index of complexity defined by low number of options and low visual complexity) and explicit learning in complex situations (Raab, 2003). Implicit learners were found to have less verbalizable knowledge that could have been used for top–down processes in contrast to explicit learners. These experiments have been ecologically validated in more realistic sports situations using different kinds of instructions (Raab and Masters, 2009). Finally, hybrid learning, as predicted, has been found to outperform more implicit or explicit learning only in complex situations, indicating a more consolidated interaction of bottom–up and top–down processes (Raab et al., 2005).

## CONCLUSION

Situation Model of Anticipated Response consequences in tactical decisions – extends and revises a previous situation model of anticipated response consequences of tactical decisions. This extension considers situation in a model of top–down and bottom–up processes and therefore indicates when specific sensorimotor interactions may occur and change behavior. Further, the model reconsiders the benefits of implicit, explicit, and hybrid learning strategies and how they may foster the use of top–down and bottom–up processes. This model has been tested mainly on sensorimotor interactions in quite complex situations, but further evidence has been found in fine motor control (Raab et al., 2013) and neurophysiological correlates are yet to be further tested (Hill and Raab, 2005). Future research should test the model’s predictions in other domains and compare the model to others available (Glöckner et al., 2012).

## Conflict of Interest Statement

The author declares that the research was conducted in the absence of any commercial or financial relationships that could be construed as a potential conflict of interest.
